# Temporary prostheses following implant surgery: A narrative review

**DOI:** 10.34172/japid.026.3871

**Published:** 2026-06-09

**Authors:** Alireza Etezadinia, Maryam Jahangiri, Hossein Salehivaziri

**Affiliations:** ^1^Department of Periodontics, Faculty of Dentistry, Shahed University, Tehran, Iran; ^2^Department of Prosthodontics, Faculty of Dentistry, Shahed University, Tehran, Iran; ^3^Department of Clinical Education, Straumann Group North America, Andover, MA, USA

**Keywords:** Computer-aided design, Dental implants, Implant-supported dental prosthesis, Osseointegration, Temporary dental prosthesis, Three-dimensional printing

## Abstract

This narrative review synthesizes evidence on temporary prostheses following implant surgery, integrating surgical timing (immediate/early vs. delayed placement and loading), primary stability and bone quality determinants, and restorative choices (prosthesis types, abutments, materials, digital fabrication including 3D printing) by searching PubMed and Web of Science (2000–2024) using MeSH and free-text terms related to implants, loading protocols, and provisionalization; five pre-2000 landmark papers provided historical context. Ninety-six human studies (clinical trials, cohorts, systematic reviews;≥6-month follow-up) met inclusion criteria. In selected cases, immediate loading achieved implant survival comparable to delayed loading, with small differences in early marginal bone loss (≈0.1–0.3 mm at 1 year). Papilla preservation relied primarily on adjacent bone morphology and 3D implant positioning. Provisional restorations critically shaped the emergence profile and peri-implant soft tissues. Digital workflows offered efficient, clinically acceptable provisionals but required investment, training, and guide accuracy. Evidence favors protocol-driven case selection and occlusal control for optimal outcomes.

## Graphical Abstract

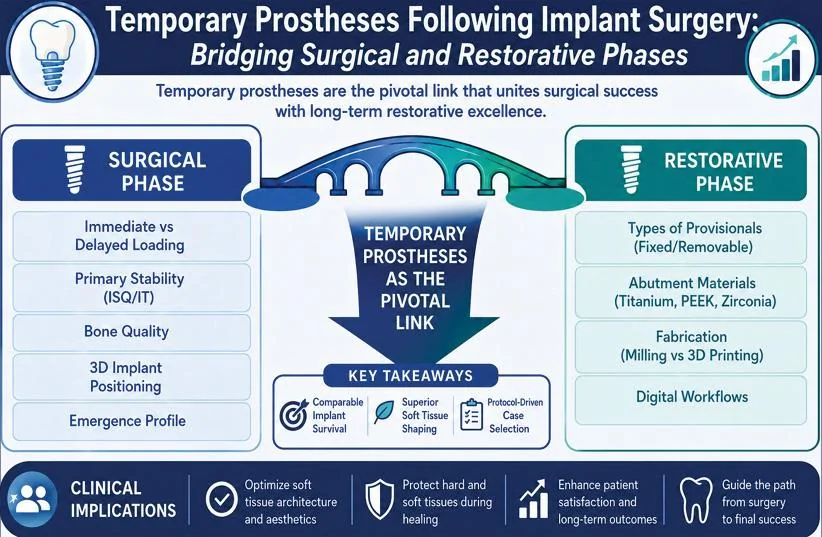

## Introduction

 Tooth loss is one of the main factors affecting patients’ oral function, esthetics, and overall quality of life.^1^ The primary causes of edentulism include caries, periodontal disease, and trauma.^2,3^ Loss of teeth compromises mastication, phonetics, and facial appearance,^1^ and when located in the anterior region, it can significantly affect social interactions and self-confidence.^4-7^

 Treatment options for replacing missing teeth depend on multiple factors, such as the patient’s age, the number and location of missing teeth, bone quality and quantity, esthetic expectations, and financial capacity.^8^ These options include fixed tooth-supported prostheses (bridges or resin-bonded bridges), removable dentures, and implant-supported fixed or removable restorations.^8^ Implant-supported fixed prostheses have become a predictable and well-documented treatment for both single and multiple tooth replacements.^8^ Continuous progress in prosthetic materials, implant surface technology, digital planning tools, and surgical techniques has further improved the long-term success of implant therapy.^9^

 Temporary (provisional) prostheses play a crucial role during the healing phase between implant placement and delivery of the definitive restoration. They preserve esthetics and occlusal function, protect surgical sites, and most importantly, guide soft tissue maturation. Properly contoured provisional restorations support the papilla and shape the emergence profile, and the key factors influencing peri-implant tissue stability and long-term esthetic outcomes.^10-18^ They also improve phonetics and patient comfort, contributing to psychological well-being and treatment acceptance.^13-18^

 Several contemporary implant placement and loading protocols have been developed to shorten treatment duration and improve outcomes.^19^ Immediate loading, defined as the placement of a prosthesis within 72 hours after implant insertion,^20^ has been widely investigated as a means to reduce recovery time and enhance patient satisfaction.^21^ When adequate primary stability is achieved, immediate loading protocols can achieve success rates comparable to conventional delayed loading, with osseointegration rates exceeding 95% in many clinical studies.^22,23^ Success depends on surgical precision, implant stability, bone density, occlusal control, and appropriate patient selection.^24^ In complex conditions, such as bone grafting sites or patients with systemic risk factors like diabetes, osteoporosis, or heavy smoking, delayed loading and cautious provisionalization remain advisable.^24,25^

 Although numerous studies have addressed surgical and restorative aspects of implant therapy, most have examined these components in isolation. Existing reviews have primarily focused on loading timelines and implant survival, or on isolated restorative elements such as mechanical fit and material properties, but have rarely addressed the critical role of temporary prostheses during the healing phase, in which surgical placement decisions directly influence restorative soft tissue management and long-term esthetic predictability. Several reviews published between 2020 and 2024 support this observation, reporting limited holistic analyses of provisional-driven tissue conditioning and workflow efficiency.^1,10-12,15,16^

 Therefore, this narrative review aims to bridge these surgical (e.g., immediate versus delayed loading, bone quality, and primary stability) and restorative perspectives (e.g., prosthesis types, abutment materials, fabrication methods, and digital workflows) by synthesizing how temporary prostheses serve as the pivotal link for optimizing peri-implant health, soft tissue stability, and overall treatment success.

 This review highlights the clinical significance of temporary prostheses for periodontists and implant specialists, emphasizing peri-implant soft tissue stability, esthetic predictability, and treatment workflow optimization.

## Search Strategy

 This narrative review was conducted using standard qualitative synthesis methods and PRISMA principles to enhance transparency and reproducibility.

 Electronic searches were performed in PubMed and Web of Science (ISI) databases for studies published between January 2000 and December 2024. The search strategy combined MeSH and free-text terms, including “Immediate Dental Implant Placement,” “Immediate Dental Implant Loading,” “Dental Implants,” “Provisional Prosthesis,” and “Implant-Supported Prostheses,” using Boolean operators AND/OR. Five seminal pre-2000 studies were also reviewed to provide historical context for tooth loss classification and early implant loading concepts.

###  Eligibility Criteria

 Studies were included if they met the following conditions:

- Published in English and conducted on human adults (≥ 18 years)
- Reported data on temporary prostheses, implant loading protocols, or related clinical outcomes, including implant survival, marginal bone loss, soft tissue response, or esthetic results
- Represented clinical trials, prospective/retrospective cohort studies, or systematic reviews with at least 6 months of follow-up

###  Exclusion Criteria

- Animal or in vitro studies
- Mini or zygomatic implants
- Case reports with inadequate data
- Non-peer-reviewed materials such as abstracts, letters, or conference proceedings

###  Study Selection and Data Extraction 

 The database search yielded 918 records (PubMed, n = 431; Web of Science Core Collection, n = 487) prior to duplicate removal. After removing duplicates (n = 86), 832 records remained for title and abstract screening. Following this screening, 623 records were excluded. Full-text assessment was performed for 209 articles, of which 113 were excluded due to failure to meet inclusion criteria. Ultimately, 96 publications were included in the qualitative synthesis, comprising 91 eligible human studies published between 2000 and 2024, identified through the electronic database search, and 5 landmark pre-2000 articles selected independently of the database search to provide historical and conceptual context.

 Study selection was performed independently by two reviewers. Disagreements were resolved through discussion and, when necessary, consultation with a third reviewer. Extracted data included study design, sample size, implant site and protocol, loading strategy, follow-up duration, outcome measures, and principal findings. A flow diagram illustrating the study selection process is provided as Figure 1.

**Figure 1:**
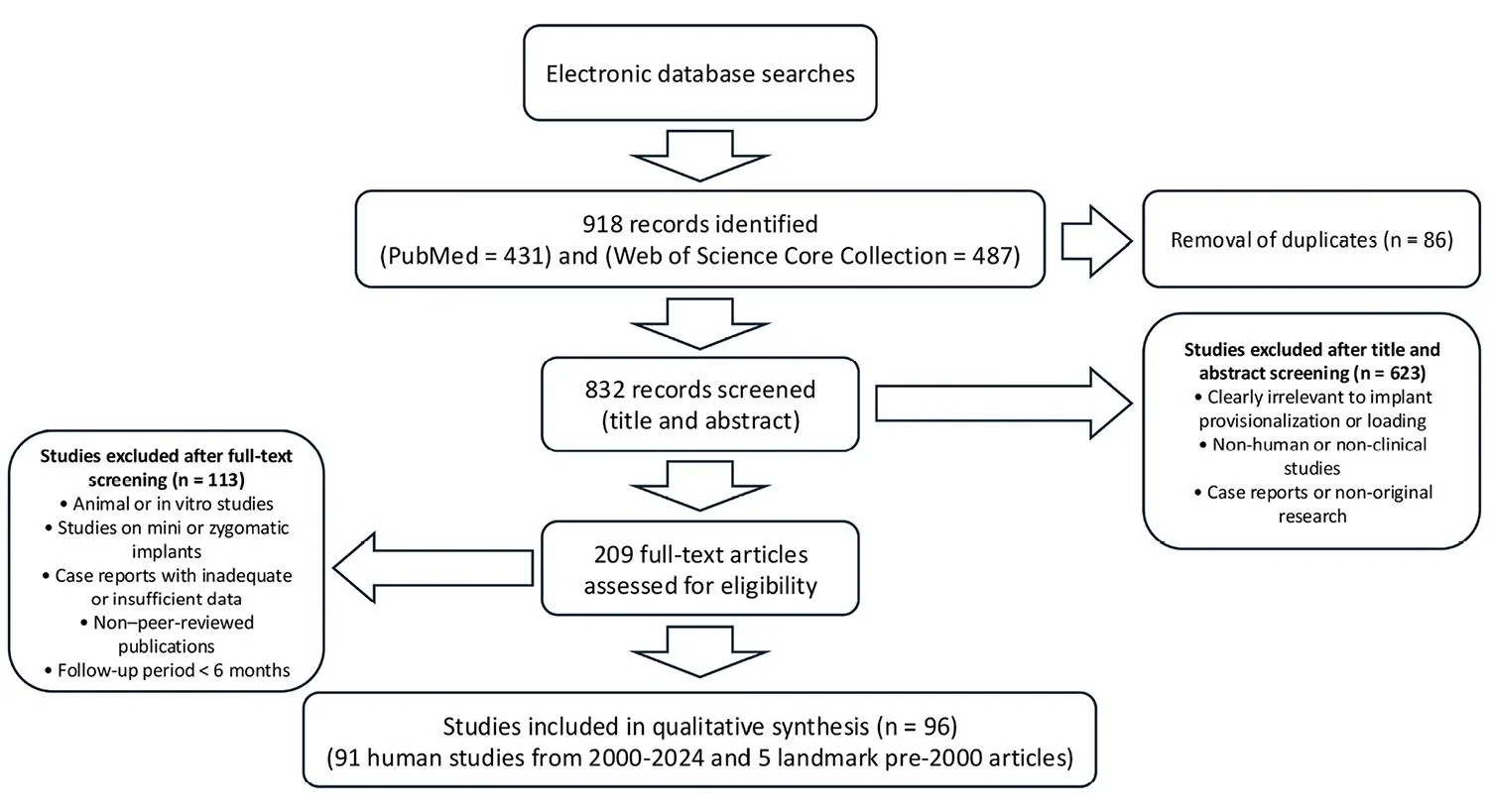
Flowchart of the literature search and study selection process

###  Organization of Findings

 Included materials were categorized into two major dimensions, surgical and restorative, for structured synthesis.

- The surgical phase section examined protocols for immediate and delayed implant placement, initial stability, loading criteria, bone quality, and the role of temporary prostheses in preserving esthetics and the emergence profile.
- The restorative phase section discussed types of temporary prostheses and abutments, material selection, fabrication techniques (milled and 3D-printed), and torque recommendations for temporary abutments.

###  Analytical Approach 

 No quantitative or statistical meta-analysis was performed, as this review aimed to provide a qualitative synthesis of evidence. Critical appraisal of study quality and evidence strength was conducted qualitatively throughout the Results and Discussion, using an adapted hierarchy of evidence that prioritized higher-level study designs (e.g., randomized trials and systematic reviews). Formal risk-of-bias tools were not applied due to the narrative scope and heterogeneity of the included literature.

###  Ethical Considerations 

 Ethical approval was not required, as the review involved previously published studies and no human or animal subjects.

###  Table Referencing 

 All tables and summary charts include numerical superscript references within their footnotes.

## Surgical Phase

###  Immediate Implant Placement and Loading Protocols 

 The following timing definitions are commonly used.^19,25-27^

- Immediate implant placement: same day as extraction
- Early implant placement: 1–4 months after extraction
- Delayed implant placement: ≥ 6 months after extraction
- Immediate loading: provisional or definitive prosthesis within 1 week of placement
- Early loading: 1 week–2 months
- Delayed loading: > 2 months

When biologic prerequisites are met (adequate bone, atraumatic extraction, ability to secure primary stability), immediate/early placement and immediate/early loading can achieve outcomes comparable to delayed protocols.^19,28^ Aiquel et al.^29^ reported a survival rate of 95–98% for both immediate and delayed loading, with marginal bone loss (MBL) of ≈0.5–1.2 mm at 1–4 years.^29^ Critical appraisal: Many included studies had modest sample sizes and heterogeneous protocols, which limits external validity.^29^

###  Factors Influencing Immediate Loading Feasibility

####  Primary Stability

 Primary (mechanical) stability at placement is a key predictor of successful osseointegration and early function.^30^ It depends on bone anatomy/quality, implant macro-design, and surgical technique.^31^ Stability tends to be higher in the mandible than in the maxilla.^32^ Excess micromotion increases the risk of early failure; higher primary stability improves predictability of immediate loading.^33^

 Quantitative notes used clinically: Many protocols proceed to immediate loading at ISQ (RFA) ≳65–70 and/or IT ≥ 30–35 Ncm, but thresholds vary by system and case. These thresholds are widely used in clinical protocols; evidence remains heterogeneous across studies.

 Critical appraisal: Observational designs and operator/device variability limit cross-study comparability.

####  Stability Assessment (IT, RFA, Periotest)

 Chairside measures include insertion torque (IT), resonance frequency analysis (RFA), and Periotest.^34^ IT is influenced by implant design and bone density and is not an absolute predictor of MBL or failure.^35^ A systematic review found no consistent association between higher IT (≈50 Ncm) and increased MBL relative to lower/moderate IT.^36^ RFA provides a non-invasive baseline; serial increases in ISQ suggest maturation of the implant–bone interface.^37,38^ Periotest values (−8 to +50) reflect mobility but have variable reliability due to multiple confounders.^37,39^

 Critical appraisal: Heterogeneity in devices, timing, and cutoffs complicates pooled interpretation.

####  Bone Quality and Site Preparation 

 Low bone density compromises primary stability and survival.^40^ Failures are more frequent in type IV bone (≈35%) than in types I–III (≈3%).^41^ Nonetheless, osteoporosis/osteopenia is not an absolute contraindication.^42^ Technique modifications, undersized osteotomy, osseodensification, step osteotomy, lateral condensation and design changes (thread geometry, length/diameter) can improve primary stability.^43-48^ Increased length and/or diameter increase contact area and may enhance osseointegration.^48^

 Critical appraisal: Evidence spans in vitro/bench studies, retrospective cohorts, and small clinical series; effect sizes depend on jaw, site morphology, and implant system.

###  Immediate versus Delayed Placement/Loading: Indications, Outcomes, Trade-offs 

 Indications for immediate placement include avulsion where repositioning is impossible, non-restorable caries/fractures, root resorption, and failed endodontic therapy. Contraindications include inability to achieve primary stability (e.g., major wall defects, poor bone quality), acute infection/active periodontitis at the site, esthetic zones requiring staged augmentation, and unfavorable root angulation.^49-52^

 When biologic and mechanical prerequisites are met, immediate loading reduces treatment time and surgical stages, with survival comparable to that of delayed loading.^21,52,53^ A meta-analysis reported first-year MBL lower by ~0.1–0.3 mm with immediate versus delayed loading.^52^ Patient-reported outcomes frequently favor immediate protocols due to shorter edentulous periods.

 Critical appraisal: Several contributing studies were retrospective and/or from high-experience centers, introducing selection and performance bias; occlusal control and parafunction are potential confounders.

 In fresh sockets, increasing the gap between the implant and socket wall reduces coronal bone-to-implant contact (BIC) and shifts it apically; nonetheless, selected immediately loaded cases may show higher BIC and less bone loss than their delayed counterparts when stability and occlusion are controlled.^54^

 Critical appraisal: Findings derive from controlled conditions; generalization to grafted, thin-walled, or parafunctional cases should be approached with caution.

###  Surgical Considerations Specific to Immediate Loading

 Immediate loading (≤ 72 h) has been associated with ≥ 95% osseointegration in appropriately selected cases.^20,22,23^ Key steps include atraumatic extraction, 3D implant positioning respecting the buccal plate, copious irrigation during osteotomy, avoidance of buccal plate perforation, grafting of residual gaps when indicated, and non-functional occlusion of the provisional during early healing.^51,55^ Systemic risk factors (e.g., uncontrolled diabetes, heavy smoking, immunodeficiency, malnutrition) argue for cautious or delayed loading.^24^

 Critical appraisal: Most evidence consists of prospective cohort studies and case series; definitions of “non-functional” loading and occlusal schemes vary, limiting comparability.

###  Interdental Papilla with Immediate Loading 

 Papilla height correlates primarily with the proximal bone crest of the adjacent tooth rather than with loading timeline per se.^56^ Prospective data up to ~1.5 years show no significant differences in papilla height between immediate and delayed loading.^57,58^ After extraction, bundle bone resorption results in transverse and vertical remodeling, more pronounced buccally, regardless of placement timing or technique.^59,60^

 Critical appraisal: Follow-up durations are often short- to mid-term; evidence in thin biotypes and high smile lines is limited.

###  Emergence Profile and Soft Tissue Architecture 

 The emergence profile (subgingival contour) governs peri-implant soft tissue form and esthetics.^10,11^ Three functional zones are described: esthetic (E), biologic (B), and crestal (C).^12^ Optimal 3D implant position preserves designed dimensions across E/B/C; malposition propagates hard/soft tissue discrepancies and compromises esthetics. Adequate soft tissue thickness is also critical: ≥ 2 mm vertical thickness supports crestal stability, and ~2–3 mm of horizontal thickness helps mask underlying components.^61,62^

 Critical appraisal: Evidence is largely observational and expert-driven; randomized comparisons of zone-specific contouring strategies are scarce. See Table 1 for E/B/C characteristics (abbreviations defined in the legend).

**Table 1:** Characteristics of peri-prosthetic zones in implant restorations

**Zone**	**Function**	**Preferred subgingival shaping**	**Predominant tissue contact**	**Typical vertical length**
E (Esthetic)	Supports gingival architecture and esthetics	Convex to support the gingiva and reduce plaque stagnation	Sulcular epithelium	~1 mm
B (Biologic)	Accommodates supracrestal tissue attachment/biologic width	Contour dictated by implant position and soft tissue thickness	Junctional epithelium	~1–2 mm
C (Crestal)	Maintains crestal bone stability	Straight (emergence transition without over-contour)	Connective tissue	~1–1.5 mm

“E/B/C” corresponds to the esthetic, biologic, and crestal subgingival zones used in implant restoration contour design.Data synthesized from: Gomez-Meda et al,^12^ Gervyte et al,^11^ and Ramos et al^10^

Figure 2 summarizes the interrelationship between these surgical determinants and subsequent prosthetic decision-making during provisionalization.

**Figure 2:**
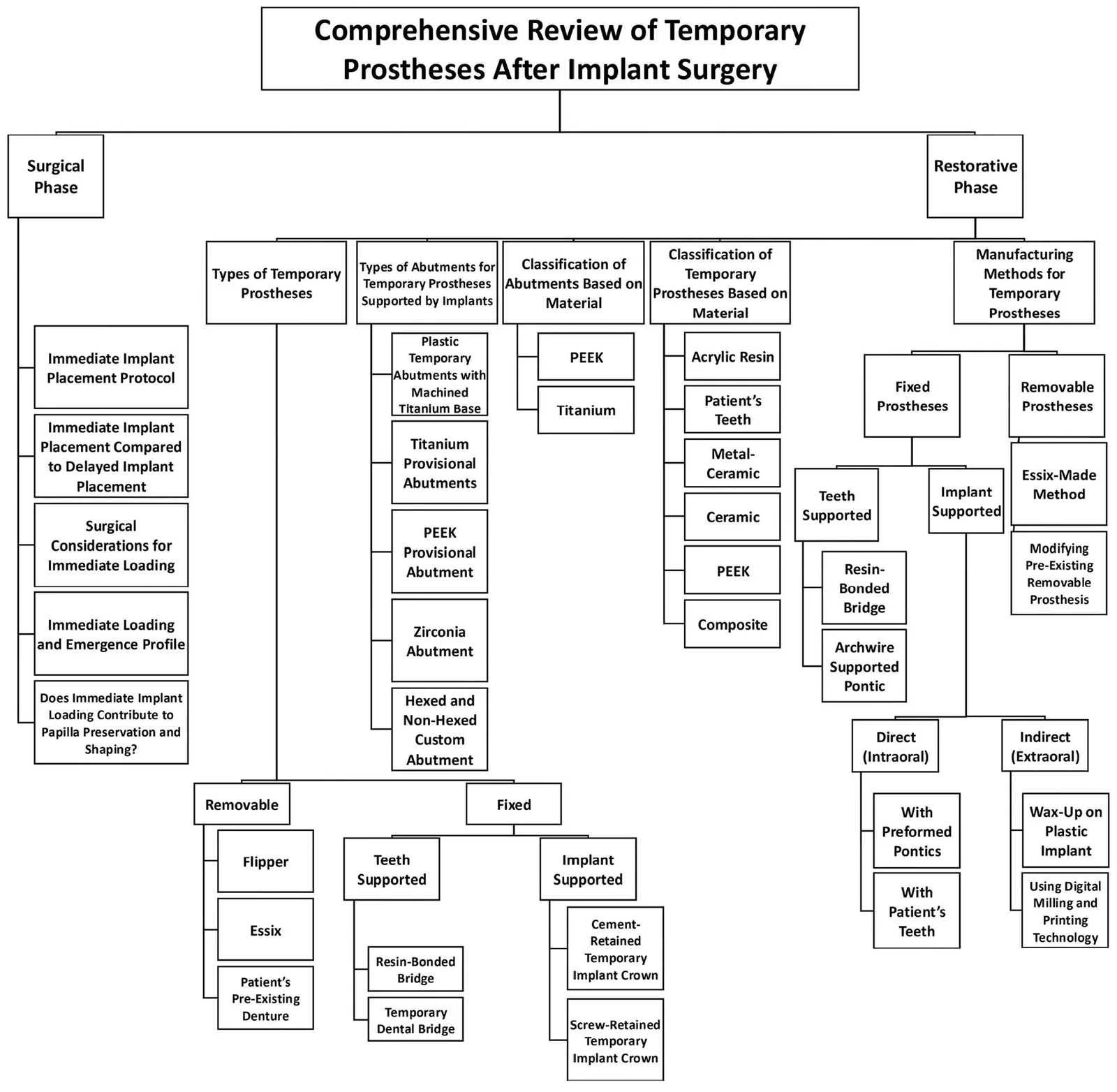
Comprehensive flowchart illustrating the interrelationship between the surgical phase (immediate implant placement protocols, primary stability, bone quality, and 3D positioning) and the restorative phase (types of temporary prostheses, abutment materials, classification of provisionals, and manufacturing methods including digital workflows)

## Restorative Phase

###  Types of Temporary Prostheses

 Temporary prostheses are broadly removable or fixed (Figure 2).^13-16,17,18^ Each option balances chair time, emergence profile control, cost, and patient comfort (Tables 2 and 3).

**Table 2:** Comparison of temporary prosthesis options (removable, tooth-supported fixed, implant-supported)

**Temporary prosthesis**	**Advantages**	**Disadvantages**	**Contraindications (typical)**	**Indications (typical)**	**Key clinical points**
Removable prosthesis (flipper/partial denture)	Easy and fast to fabricate; low cost; easy to adjust/add teeth	Risk of contacting/scraping surgical site; may compromise grafts if it impinges; esthetics/comfort variable	Strong gag reflex; sites requiring strict GBR protection	Pre-surgery, post-surgery during osseointegration, long edentulous spans	Do not allow the base to contact surgical tissues; relieve over grafts; reline as tissues remodel.^13,17,63^
Essix appliance (vacuum-formed retainer with pontic)	Low cost; quick; minimal tooth prep	Does not shape emergence profile; accelerated wear; chewing limitations; high plaque accumulation	Long-term use where durability/occlusion is needed	Limited occlusal space; anterior esthetics, deep bite scenarios	Ensure no tissue contact over the surgery site; emphasize hygiene; short-term only.^15,16^
Fixed tooth-supported (bridge, resin-bonded, archwire-supported pontic)	Can shape soft tissue/emergence; better function/esthetics; more stable	Higher cost; requires suitable abutments or orthodontic anchorage	No suitable adjacent teeth; long spans without abutments	Esthetic zone with adjacent teeth; when avoiding a removable appliance	Use brackets/archwires/fiber to carry composite/acrylic or natural-tooth pontic; protect surgical site.^13,14,64^
Fixed implant-supported (interim on implant[s])	Patient comfort; fixed esthetics/function; emergence/papilla shaping	Higher cost; requires primary stability	Insufficient primary stability; immediate grafted single-site needing protection	Immediate loading cases meeting stability criteria	Keep out of occlusion if interim nonfunctional; verify torque per system.^16,65,66^

The table contrasts common provisional choices during immediate/early implant therapy regarding clinical use and risks. Abbreviations: GBR: guided bone regeneration. Data synthesized from: Siadat et al,^13^ Santosa,^14^ Field & Wassell,^15^ Eachempati & McColl,^16^ Cho et al,^17^ Kan et al,^18^ St George et al,^63^ Darbar et al,^64^ Mijiritsky,^65^ and Dumbrigue et al^66^

**Table 3:** Abutment options for implant-supported interim prostheses: Summary of properties and uses

**Abutment type**	**Advantages**	**Disadvantages/limitations**	**Typical applications**
Plastic temporary abutment with machined titanium base	Inexpensive; chairside adjustability; no heat during intraoral trimming	Limited for large angulations depending on the system	Screw-retained or cement-retained provisionals; rapid chairside fabrication.^65-69^
Titanium provisional abutment	Widely available; durable; cost-effective; easy to use	Typically cement-retained only; esthetic shine-through in thin tissue	Cement-retained interim crowns/bridges.^69,70^
Custom abutment (hexed/anti-rotational or non-hexed/rotational)	Best fit with angulation issues and tissue height variation; long-term capable	Higher cost; lab-intensive; requires planning	Screw- or cement-retained; use for complex emergence and alignment control.^68,69,71^
Zirconia abutment (1- or 2-piece with Ti base)	Superior esthetics; stable soft-tissue color	Risk of chipping/fracture (esp. 1-piece); technique-sensitive	Screw- or cement-retained in esthetic regions; prefer Ti-base designs.^72,73^
PEEK provisional abutment	Tooth-like color; MRI compatible; low modulus may cushion load	Inferior mechanical strength vs Ti; risk of veneer debonding	Short-term use in esthetic zones with low occlusal load.^74-76^

“Hexed/anti-rotational” prevents rotation for single units; “non-hexed/rotational” allows seating for certain multi-unit scenarios (verify system specifics). Abbreviations: PEEK: polyetheretherketone; Ti: titanium.Data synthesized from: Hsiao et al,^67^ Agustín-Panadero et al,^68^ Al-Thobity,^69^ Lee et al,^70^ Huang & Wang,^71^ Amorfini et al,^72^ Kammermeier et al,^73^ Najeeb et al,^74^ Suphangul et al,^75^ Santing et al^76^

1. Removable provisionals (flippers, Essix).^13-17^ A pre-existing removable prosthesis can be relined with a soft liner.^17^ Typical advantages are low cost and rapid fabrication/adjustment.^13,14,16,17^ Limitations include no emergence profile shaping,^18^ risk of pressure on grafted/surgical sites if not relieved,^17^ and plaque accumulation (notably with Essix), necessitating strict hygiene.^16^ Essix appliances are short-term options due to occlusal wear, compromised esthetics, and limited chewing.^15^ After surgery, ensure no tissue contact; when delivered immediately, continuous wear for 24–48 h helps prevent inflammatory swelling that can prevent proper seating.^16,63^

Critical appraisal: Evidence is largely case series and expert protocols; patient adherence and hygiene are key confounders.

2. Fixed tooth-supported temporaries (resin-bonded, archwire-supported, provisional bridges).^13,14,17^ Appropriate when adjacent teeth can support a pontic (e.g., anterior cases in younger patients with sensitivity concerns).^64^ Properly designed provisionals shape peri-implant soft tissue at the surgical site.^17^

Critical appraisal: Indications are well described, but comparative trials against implant-supported provisionals for soft tissue metrics are limited.

3. Fixed implant-supported provisionals (provisionals on the implant). These can preserve hard/soft tissues, facilitate emergence profile and papilla conditioning, and improve comfort and esthetics during healing.^16,65,66^

Quantitative note: Where reported, early function shows survival comparable to delayed loading with MBL differences ≈0.1–0.3 mm in year 1, favoring immediate loading in selected cases.^52^

 Critical appraisal: Outcomes depend on primary stability, occlusal control, and biotype; many studies are single-center cohorts.

###  Abutments for Implant-supported Provisionals 

 Available options include plastic provisional abutments with machined titanium bases, titanium provisional abutments, PEEK provisional abutments, and custom (titanium/zirconia) abutments (Figure 2).^67,68^

- Titanium provisional abutments: common, cost-effective, straightforward; typically cement-retained only.^69,70^
- Plastic provisional abutments: inexpensive; intraoral trimming without heat; usable for screw- or cement-retained provisionals.^65,69^
- Custom abutments (Ti/Zr): indicated for angulation, variable tissue height, or esthetic demands; strongest mechanical performance and long-term suitability; higher cost/lab time.^68-71^
- Zirconia abutments: esthetic, in one-piece or two-piece with Ti base; wear/fracture risk higher than titanium in posterior load.^72,73^
- PEEK provisional abutments: favorable handling/biologic properties; mixed mechanical data (similar strength to Ti in one study; weakest in another lab study).^68,74-76^

Critical appraisal: Much of the comparative mechanical evidence is in vitro; clinical validation is limited and system-specific.

###  Recommended Abutment Torque for Provisionals 

 Reported values vary by implant system and component: 15–20 Ncm is commonly used for provisional abutments.^13,77-79^

- Castellon et al.^77^: 20 Ncm
- Makarov et al.^78^ (Straumann): 15 Ncm
- Sandoval-Guevara et al.^79^ (Nobel Biocare): 15 Ncm

Critical appraisal: Heterogeneity in designs and screws precludes a universal torque; follow manufacturer specifications and consider screw size/material.

###  Abutment Materials (Titanium vs. PEEK; Zirconia Noted Above) 

 Titanium (commercially pure or Ti alloys) remains the standard for strength, corrosion resistance, and interface precision; potential gray show-through in thin tissues is an esthetic limitation.^77,78^

 PEEK offers a lower elastic modulus, radiolucency, and favorable handling/biologic behavior; however, mechanical performance varies across studies.^74-76^ Clinical translation: PEEK is reasonable for short-term esthetic zones but should be avoided in high-stress situations.^68,74-76^

 Critical appraisal: Most data are bench/case series; high-quality head-to-head clinical trials are scarce.

###  Temporary Prosthesis Materials (PMMA, Composite, Metal-Ceramic/Ceramic, PEEK, Natural Tooth)

- PMMA vs. UDMA: PMMA favored for chemistry/handling; UDMA for faster fabrication and possibly periodontal friendliness.^80^
- PEEK provisionals: clinically acceptable strength with occasional veneer/pink resin debonding manageable chairside.^81^
- Natural-tooth options: use of an extracted crown in Essix^82^ or as an implant provisional (or using the prior all-ceramic crown) is feasible with proper bonding protocols.^83^

Critical appraisal: Most reports are case series/case reports; follow-up is often short-term; standardized outcome measures (MBL, PES/WES, PROMs) are reported inconsistently. Table 4 summarizes key outcomes across studies.

**Table 4:** Summary of key clinical outcomes from included studies

**Outcome**	**Range/Estimate**	**Context/Notes**	**References**
Implant survival rate	95–98%	Comparable between immediate and delayed loading in selected cases; 1-4 years of follow-up	Aiquel et al,^29^ Eini et al,^52^ Singh et al.^53^
Marginal bone loss (MBL)	0.5–1.2 mm at 1–4 years; 0.1–0.3 mm less with immediate loading in year 1	Small early differences favoring immediate in meta-analyses; heterogeneous protocols	Aiquel et al,^29^ Eini et al.^52^
Primary stability thresholds (clinical notes)	ISQ ≥ 65–70; IT ≥ 30–35 Ncm	Variable by system; used for immediate loading decisions	Szmukler-Moncler et al,^33^ Norton,^35^ Roca-Millan et al,^36^ Swami et al,^37^ Aparicio et al.^38^
Failure rates by bone type	≈35% in type IV bone; ≈3% in types I–III	Influenced by density, not absolute contraindication for osteoporosis	Busenlechner et al,^40^ Jaffin & Berman,^41^ Holahan et al.^42^
Complications (mechanical/technical)	Occasional veneer debonding (PEEK); wear/fracture (zirconia posterior); low events in milled workflows (e.g., 1 loss/55 implants at 1 year)	System-specific; higher in high-load or poor stability cases	Amorfini et al,^72^ Kammermeier et al,^73^ Najeeb et al,^74^ Suphangul et al,^75^ Santing et al,^76^ Makarov et al,^78^ Kwan & Kwan^81^

The table summarizes extracted quantitative data on survival, bone loss, and complications across loading protocols and provisional types. Abbreviations: ISQ: implant stability quotient; IT: insertion torque

###  Fabrication Methods 

 Removable provisionals: Essix formed on a model; can incorporate extracted natural teeth to enhance esthetics.^82^

 Fixed implant provisionals

- Castellon et al.^77^ describe four pathways: (1) pre-surgical prep of abutment/crown with guided placement; (2) chairside abutment + immediate impression and laboratory-made provisional; (3) chairside modification of prefabricated abutment and pickup with acrylic; (4) one-piece systems with 2 mm of occlusal clearance (not for angled needs).^77^
- Milled same-day full-arch workflows with stabilizing pins and 15 Ncm re-fixation have shown 1-year functionality with low mechanical events (one implant loss in 55).^78^
- Decision aids can prioritize options (e.g., time to delivery, need to avoid occlusal contacts) and often select Essix when no functional loading is desired.^84^
- Conversion of existing dentures to screw-retained interim prostheses is achievable; use of PEEK fasteners may reduce later fracture risk.^85^
- Six practical methods (pre-index/acrylic, polycarbonate shell, reuse of prior crown, reuse of laminate, use of extracted crown facade, lab composite) are detailed for single-unit situations.^83^

Critical appraisal: Protocols are well described but primarily observational; outcomes depend on occlusal control and soft-tissue biotype.

###  Printing vs. Milling for Provisionals 

 Across studies, both printed and milled provisionals achieve clinically acceptable properties; relative performance varies by resin and workflow.

- Fracture strength: printed > milled/self-cured in one study;^86^ milled ≥ printed in another 2024 study; both acceptable.^87^
- Wear: self-cured shows highest wear; printed surfaces smoother after wear than milled.^88^
- Marginal fit: printed and milled superior to self-cured.^87^
- Color stability and surface hardness/plaque: self-cured PMMA shows the greatest color change and highest plaque; printed lowest color change and plaque in the same study; sealants are recommended for color stability over time.^89^

Critical appraisal: Test conditions differ (printers, resins, post-cure, CAM parameters); direct clinical generalization should be approached with caution.

###  Digital Dentistry and Immediate Loading 

 Digital workflows (intraoral scanning, CAD/CAM, guided surgery, and print/mill) can reduce fabrication time and costs compared with conventional methods for both single- and multi-unit implant restorations.^90^ For single crowns, especially posterior implants, fully digital, monolithic restorations on prefabricated Ti bases are robust.^91,92^ Guided approaches in immediate placement rely on an accurate tooth-supported fit; tooth mobility, scan/manufacture errors, and large support surfaces can increase deviations, while selective support points and metal guides may improve stability.^93,94^

 All-on-4 protocols leverage CBCT-based planning and same-day provisionalization (steps outlined in Results–Surgical), offering fewer grafts, shorter treatment duration, and faster recovery.^95^

 Critical appraisal: Most digital evidence comes from prospective cohort studies with center-level experience; randomized trials on soft tissue/esthetic endpoints remain limited.

## Summary of Evidence and Clinical Implications

 This review underscores practical pathways for provisionalization in implant therapy, guiding clinicians toward evidence-based decisions that optimize peri-implant health and interdisciplinary coordination. Within the scope of immediate/early vs. delayed loading, emergence profile management, abutment/material selection, and fabrication pathways, current evidence indicates the following.

###  Loading Protocols and Biologic Rationale

 Immediate/early loading yields implant survival comparable to delayed loading when primary stability and occlusal control are ensured; early MBL differences are small (≈0.1–0.3 mm at 1 year in selected cohorts).^20,23,29,52^ Strength of evidence: moderate.

###  Soft Tissue Outcomes and Provisional Contouring

 Papilla and emergence profile depend primarily on bone morphology, tissue thickness, and implant position; immediate provisionals enable soft tissue shaping but do not overcome unfavorable anatomy.^56-62^ Strength: moderate for determinants; low for loading effect per se.

###  Material and Abutment Selection

 Titanium (stock/custom) remains the mechanically reliable choice; zirconia improves esthetics but carries a higher fracture risk in posterior loads; PEEK is acceptable for short-term use in low-load esthetic zones, given mixed mechanical data.^68-76^ Strength: low to moderate.

###  Provisional Materials

 PMMA/UDMA each has advantages; reused natural crowns/teeth are viable with proper protocols.^80-83^ Strength: low (short-term, nonrandomized).

###  Manufacturing Workflows: Printing vs. Milling

 Both can deliver clinically acceptable provisionals; superiority is material/workflow-dependent; self-cure is least favorable for wear/color/plaque.^86-89^ Strength: low to moderate (in vitro-dominant).

###  Digital Workflows: Benefits and Limitations

 Digital pathways reduce time/cost and support same-day provisionalization; limitations include capital/maintenance costs, a learning curve, and potential inaccuracies in guidance in specific scenarios.^90-96^ Strength: moderate for efficiency; low for long-term biologic/esthetic endpoints.

###  Practical Implications Guide Decision Pathways during Provisionalization

- Prioritize primary stability metrics (e.g., RFA trend over time, insertion torque per system guidance) and strict occlusal control when considering immediate loading.^33^
- In the esthetic zone, plan 3D implant position and soft tissue thickness targets (> 2 mm vertical; ≈2–3 mm horizontal) before relying on provisional contouring.^61,62^
- Choose abutment/material based on load, esthetics, and retrievability: Ti/custom in higher load; Zr/Ti-base in high-esthetics; PEEK for short-term esthetic situations.
- When adopting printed provisionals, use validated resins and manufacturer-specified post-cure; avoid self-cure for longer service due to wear/color/plaque risks.
- For digital workflows, budget for training/calibration and consider selective support or metal guides when tooth mobility or large spans risk guide misfit.

## Discussion

 This narrative review synthesized surgical and restorative considerations for temporary prostheses after implant surgery, with particular attention to loading protocols, emergence profile management, abutment/material selection, and analog-to-digital fabrication pathways.

###  Why Do Immediate and Delayed Loading Show Similar Outcomes?

 Across systematic reviews and consensus statements, implant survival and early marginal bone loss (MBL) are comparable between immediate/early and delayed loading when case selection is appropriate and primary stability is achieved.^20,23,29,53^ Biologically, this equivalence likely stems from the bone’s adaptive remodeling capacity: as long as micromotion remains below fibrotic thresholds (~150 μm), osseointegration proceeds via secondary stability development, even under controlled functional loads;^33,38^ delayed protocols may allow more initial healing but do not inherently result in superior outcomes in low-risk cases due to similar inflammatory and osteogenic responses.^20,23^ Clinically, standardized drilling, under-preparation in softer bone, and use of contemporary implant designs reduce early instability.^31,43-47^ Where differences are reported (e.g., slightly lower MBL with immediate loading in select cohorts; ~0.1–0.3 mm at 1 year), the magnitude is small and likely confounded by case selection and center experience.^52^ Conflicting evidence tends to arise in low-density bone (Type IV), grafted sites, heavy smoking, or uncontrolled systemic disease, where primary stability and biologic capacity to remodel are compromised.^40-42,45-47^ In these situations, immediate loading remains less predictable.

###  Soft Tissue/Esthetic Outcomes and the Role of Provisionals

 Papilla height and emergence profile stability relate more to bone morphology and implant position than to loading per se.^56-62^ Immediate temporization facilitates subcritical/critical contouring that guides tissue form but cannot compensate for thin buccal plates or implants placed too facially.^10-12^ Evidence for papilla preservation with immediate loading is neutral in the short term (≈1–1.5 years).^57,58^ Long-term esthetic data remain limited. Taken together, provisionals are tools for shaping the soft tissue, with success contingent on underlying anatomy and 3D implant placement.

###  Abutment and Material Choices for Provisionals

 Titanium provisional and custom abutments remain the mechanical benchmark; zirconia improves esthetics but carries a higher risk of wear and fracture under posterior loads.^68-73^ PEEK offers handling and esthetic advantages but shows variable mechanical performance across bench studies.^68,74-76^ Short-term clinical use is reasonable in low-load esthetic zones. For provisional crown materials, PMMA and UDMA each offer trade-offs (handling vs. speed/periodontal friendliness).^80^ Recycling the patient’s own crown/tooth can yield highly acceptable esthetics with careful bonding protocols.^82,83^ Overall, the certainty of evidence for superiority of abutment/crown materials is low, as most data are in vitro or from case series with short follow-up.

###  Printing vs. Milling

 Both workflows achieve clinically acceptable marginal fit and fracture resistance for interim restorations; which one is “stronger” varies by study, resin, and post-processing.^86-89^ Self-cure methods are consistently inferior in wear, color stability, and plaque resistance.^88,89^ Current evidence is low-to-moderate certainty, largely in vitro, and clinicians should prioritize validated materials and correct post-cure protocols.

###  Digital Workflows, Benefits and Limitations

 Digital approaches (IO scanning, CAD/CAM, guided surgery, printed/milled provisionals) can reduce fabrication time and cost and streamline same-day delivery in eligible cases.^9,90-92^ However, barriers include capital cost, software/hardware learning curve, team training, maintenance, and potential inaccuracies from scanning/manufacturing or guide support on mobile teeth; selective support points and, when indicated, metal guides can mitigate deviations.^93,94^ Access and expertise are uneven across practices, which may partly explain heterogeneity in reported outcomes. Evidence here is moderate certainty for efficiency, low certainty for long-term biologic/esthetic superiority.

###  Limitations of This Review

 As a narrative review, we did not perform quantitative synthesis. Included studies vary in design, follow-up, endpoint definitions (e.g., survival vs. success, MBL measurement protocols), and industry support, which can introduce bias. Heterogeneity in methodologies (e.g., varying implant systems and patient cohorts) limits direct comparisons, while short follow-up in many studies (often 1–2 years) restricts long-term insights; potential reporting biases (e.g., underreporting of failures) and selection biases from high-volume centers further caution interpretation. Where possible, we highlighted ranges (e.g., survival 95–98% and MBL ≈0.5–1.2 mm at 1–4 years in mixed protocols),^29^ but these should be interpreted with caution.

###  Future Directions

 Priorities include (i) randomized or well-matched prospective studies stratified by bone quality and grafting status for immediate vs delayed loading; (ii) standardized, protocol-driven trials on emergence-profile contouring with validated esthetic indices (PES/WES) and soft-tissue thickness controls; (iii) head-to-head clinical trials comparing printed vs. milled provisionals on fracture/wear/MBL/PROMs; and (iv) implementation studies quantifying the learning curve and cost-effectiveness of fully digital provisional workflows.

## Conclusion

 Temporary prostheses play a central role in modern implant therapy by protecting surgical sites, shaping peri-implant soft tissues, and supporting esthetic and functional outcomes. Careful case selection, precise 3D implant positioning, and well-contoured provisionals remain essential for long-term success. Clinicians are encouraged to adopt protocol-driven approaches and interdisciplinary collaboration to achieve predictable results in both immediate and delayed loading scenarios.

## Competing Interests

 The authors declare no financial or non-financial competing interests. The corresponding author’s affiliation with Straumann North America (Manager of Clinical Education) had no influence on the study design, data collection, interpretation, or writing of this manuscript, and no products or services from Straumann are referenced in this article.

## Data Availability

 No datasets were generated or analyzed during the current study. All data supporting the findings of this narrative review are derived from previously published studies cited in the reference list.

## Ethical Approval

 Not applicable.
